# Climatic Variables as Drivers of *Pterocarpus erinaceus* (Fabaceae): Distribution and the Implications of Climate Change

**DOI:** 10.1002/ece3.72820

**Published:** 2026-01-05

**Authors:** İdris Sari, Bio Ismael, Fazal Ullah, Gafarou Agoundé, Faruk Yildiz

**Affiliations:** ^1^ Department of Biology, Faculty of Science and Art Erzincan Binali Yildirim University Erzincan Türkiye; ^2^ Department of Forest‐Wildlife and Ecological Engineering, Higher Institute of Environment and Ecology University of Diffa Diffa Niger; ^3^ College of Life Sciences Northwest Normal University Lanzhou China; ^4^ Le Laboratoire de Biomathématiques et d'Estimations Forestières University of Abomey‐Calavi Cotonou Benin

**Keywords:** Africa, climate change, conservation, ecophysiology, *Pterocarpus erinaceus*

## Abstract

*Pterocarpus erinaceus* is an ecologically and economically important tree species native to the Sahel region of West Africa, facing severe threats due to overexploitation, habitat degradation, and climate change. This study assesses the impact of key climatic variables on the species' current distribution and projects potential range shifts across Africa under mid‐21st century climate scenarios. Using an ensemble modeling approach that combines various algorithms and utilizes 37 comprehensive climatic variables, the analysis revealed significant patterns of highly suitable habitat, concentrated primarily in West Africa. Projections under low and medium emission scenarios for 2040–2060 and 2080–2100 periods predict a reduction in climatically unsuitable areas and a potential expansion of suitable habitats, suggesting that the species may be somewhat resilient to medium‐term climate changes. The primary climatic factors limiting the species' distribution were identified as the Mean Temperature of the Driest Quarter and the Climatic Moisture Index. These results underscore the species' ecophysiological dependence on specific temperature and moisture regimes. Crucially, while our projections suggest the species exhibits resilience and potential habitat expansion under medium‐term climate change, its realization is contingent upon mitigating persistent anthropogenic pressures. Therefore, to ensure the species' long‐term persistence and maintain the ecological integrity of the West African Savanna Biome, conservation strategies must prioritize aggressive, in situ actions focused on sustainable management, controlled harvesting, and the protection of current and future suitable habitats, rather than focusing solely on long‐term climate change adaptation measures.

## Graphical abstract

The study investigates the distribution of *Pterocarpus erinaceus* in Africa, revealing that the Mean Temperature of the Driest Quarter and the Climatic Moisture Index are the most influential climatic variables determining its distribution. These findings offer new insights into the potential impacts of climate change on the species' habitats and its ecophysiological tolerance.

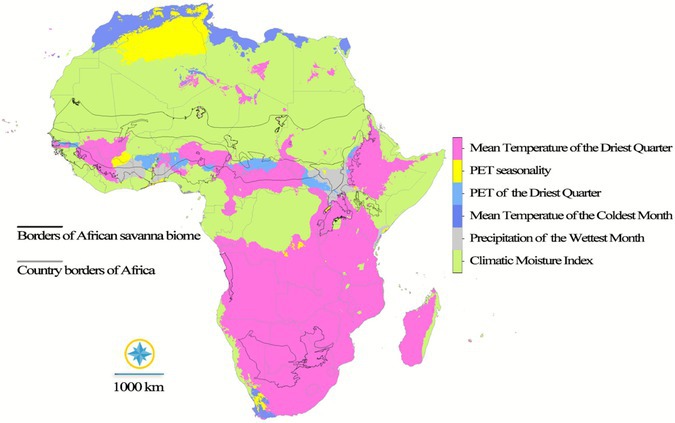

## 1 Introduction

*Pterocarpus erinaceus* Poir. is a critical multipurpose tree species native to the Sahelian region of West Africa, boasting a broad distribution that extends from southern Senegal to the western Central African Republic (Ahoton et al. 2009; Fulgence et al. 2024). This widespread legume is highly valued for its premium timber, which is a top‐traded tropical hardwood used globally in woodworking and musical instrument crafting. Beyond its significant economic value, the species serves as a vital resource for traditional medicine and livestock fodder. Ecologically, it enhances soil fertility through nitrogen fixation, and this multifaceted importance highlights its key role in the regional ecology and economy (Oteng Mintah et al. 2019; Yusuf et al. 2020; Peter et al. 2021; Alagbe et al. 2024; Aliou et al. 2024).

As a widespread species, climate stands as the primary determinant of plant species distribution worldwide. Long‐term atmospheric conditions like temperature and precipitation directly influence plant physiology, dictating survival, growth, and reproductive success. Distinct climate zones create selective pressures, allowing only those species with suitable adaptations to thrive. Consequently, a plant species' existence and distribution are largely confined to regions where climatic conditions align with its physiological tolerances and adaptive capacities (Garnier et al. 2016; Sandel et al. 2019; Cai et al. 2024; Huang et al. 2024; Li and Prentice 2024).

The widespread and adverse impacts of climate change are already acutely felt across Africa, resulting in reduced food and water security, negative consequences for human health, and significant economic and societal losses. Crucially, vulnerable African communities, who have historically contributed the least to current climate change, are disproportionately affected. The increasing frequency of extreme weather and climate events has also exposed millions to acute food and water insecurity and is increasingly driving displacement across Africa (IPCC 2023). Therefore, addressing climate change impacts necessitates integrating local ecological knowledge with global climate assessments to ensure that conservation strategies are both context‐specific and globally informed (Solecki et al. 2024).

The conservation and sustainable management of *P. erinaceus* is of paramount importance due to the significant anthropogenic pressures it faces (Barstow 2018), including overexploitation and habitat loss (Dumenu and Bandoh 2016; Adji et al. 2021; Christine et al. 2021). Despite the species' inherent resilience, the overall impact of climate change is expected to be detrimental. While existing species distribution models (SDMs) have predicted potential upward population shifts by incorporating intraspecific variation (Dimobe et al. 2020; Biaou et al. 2023), climate change is anticipated to worsen existing anthropogenic pressures like overexploitation for timber and fodder (Dimobe et al. 2020). This synergistic effect fundamentally increases the species' vulnerability. Consequently, a more nuanced understanding of how climate change will affect *P. erinaceus* 's distribution is urgently needed to inform effective conservation efforts.

Although SDMs have been extensively used, previous climate change analyses on *P. erinaceus* have often presented divergent results due to several limitations. For instance, many past studies concentrated on regional scales, focusing on assessing impacts within specific areas like Burkina Faso or Benin, which overlook the necessary continental context required for developing broad conservation strategies for such a widespread species (Dimobe et al. 2022; Biaou et al. 2023; Asigbaase et al. 2024). Furthermore, the ecological requirements and precise climatic preferences of *P. erinaceus* have not been sufficiently investigated; past modeling efforts frequently relied solely on standard climatic variables, failing to capture the complex ecophysiological processes (such as soil moisture dynamics, temperature seasonality, and stress) relevant to savanna species (Dimobe et al. 2020, 2022; Biaou et al. 2023). Finally, a methodological constraint is that some previous studies often relied on older climate scenarios (Representative Concentration Pathways), making their findings less comparable with current global climate mitigation planning, which utilizes the Shared Socioeconomic Pathways framework.

Given *P. erinaceus* ' recognized vulnerability to both intense anthropogenic pressures and the overarching threat of climate change, this study aims to elucidate its potential future. We address existing research gaps—which often involve restricted geographical scope and the use of older climate scenarios—by conducting a novel, continental‐scale ensemble modeling approach across Africa. This work advances current understanding by utilizing an expanded dataset of 37 climate predictor variables and focusing on future low‐to‐moderate emission scenarios (2040–2060 and 2080–2100). Specifically, we seek to: (1) delineate the current suitable climate areas for the species; (2) project the potential spatial shifts under these emission scenarios to identify climate resilience; and (3) determine the key climatic drivers influencing its continental distribution and persistence. Ultimately, this research furnishes critical insights to inform the prioritization of urgent conservation planning and management actions for safeguarding *P. erinaceus* against intensifying pressures.

## 2 Materials and Methods

### 2.1 Study Species Ecological Profile

*P. erinaceus* is found in open, dry forests in semi‐arid and sub‐humid lands, where it experiences an average annual rainfall of 600–1500 mm and a long dry season lasting 8–9 months. The species is highly tolerant of its natural range's average annual temperatures of 15°C–35°C, even withstanding extreme heat over 40°C. It thrives at low altitudes (0–600 m) and shows remarkable resilience to drought and savanna bushfires, allowing it to readily colonize deforested savannas and fallow lands (Ahoton et al. 2009; Tosso 2013; Kossi et al. 2021). This adaptability is further demonstrated by its morphological plasticity and tolerance to a wide range of climatic conditions (Kossi et al. 2020, 2021; Lee et al. 2023; Asigbaase et al. 2024).

### 2.2 Compiling of the Species' Occurrence Records

**FIGURE 1:**
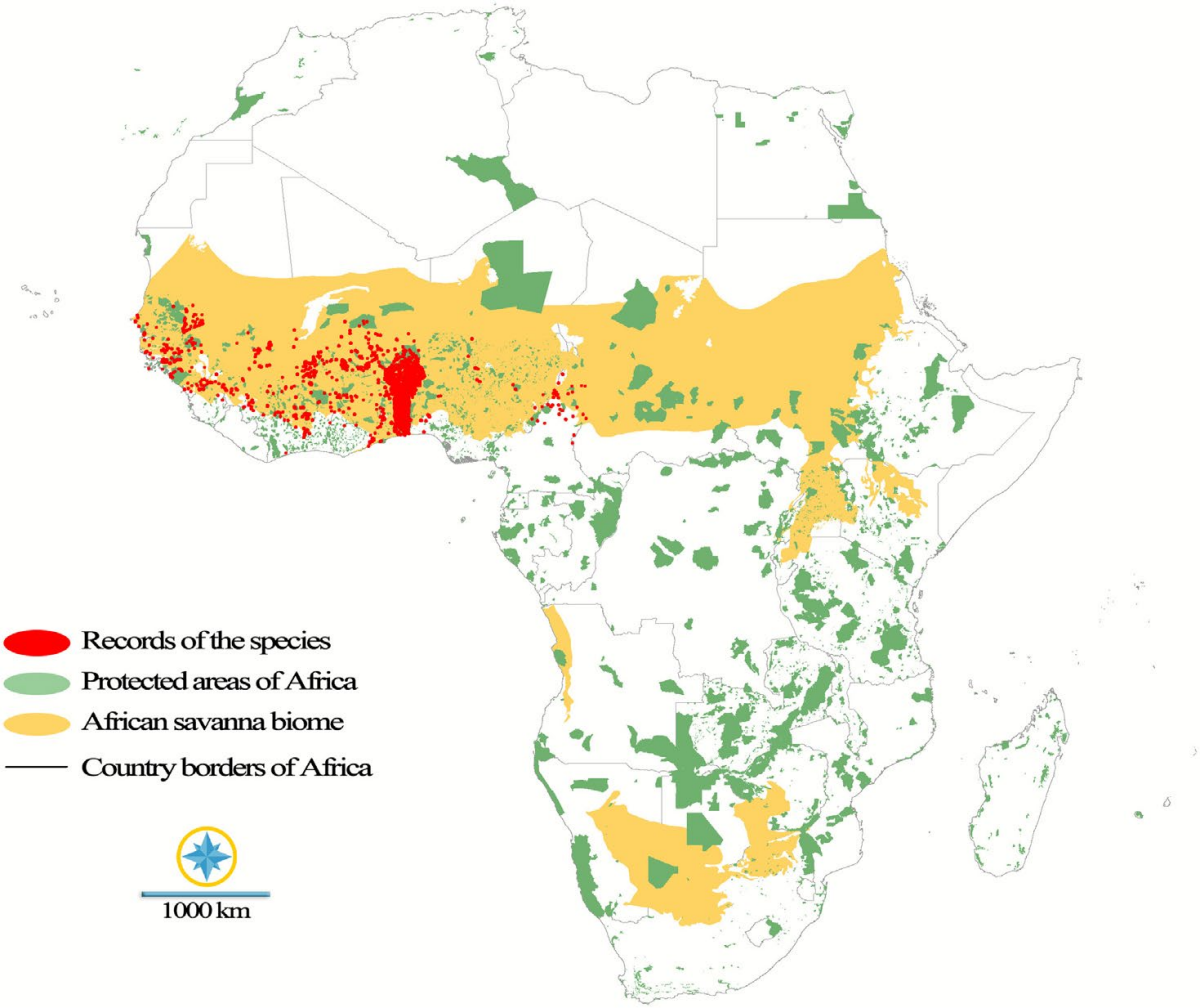
Current distribution records of *P. erinaceus* in Africa, overlaid with African protected areas and the species' predominant savanna biome.

A total of 11,797 occurrence records for the species were compiled from the Global Biodiversity Information Facility (GBIF 2025) via the occCite R package (Owens et al. 2021). To enhance the accuracy of our species distribution models (SDMs), we implemented a two‐step data cleaning process. First, obvious outliers were manually removed. Second, we employed the enmSdmX R library (Smith et al. 2023) to thin the remaining records based on a raster layer resolution of 2.5 arcminutes. This rigorous filtering resulted in a final dataset of 2220 records, which were subsequently used to develop SDMs for both the present and projected future distribution of the species (Figure 1; See Supplemental Occurrences in Appendix S1).

### 2.3 Preparation of Bioclimatic and Envirem Predictor Variables

To define the environmental space for our modeling, we utilized a comprehensive dataset of climatic variables at a 2.5 arc‐minute resolution. These variables were sourced from the WorldClim database (version 2.1) (Fick and Hijmans 2017). We used the standard 19 Bioclimatic variables directly obtained from WorldClim, which represent annual trends, seasonality, and extremes of temperature and precipitation. Additionally, to capture the complex ecophysiological processes relevant to *P. erinaceus* , we generated a suite of 18 supplementary climatic variables known as Envirem variables. These Envirem variables were calculated using the Envirem R library (Title and Bemmels 2018), utilizing WorldClim's minimum temperature, maximum temperature, and precipitation data and following the computational procedures detailed on the authors' website (ENVIREM 2025). In total, the full dataset used for initial modeling comprised 37 climatic predictor variables (19 Bioclimatic +18 Envirem), ensuring a comprehensive definition of the species' climatic niche.

### 2.4 Variable Selection Procedures

To ensure the ecological relevance and statistical robustness of our predictive models, we implemented a variable selection process to mitigate multicollinearity and variance inflation (Dorman et al. 2012; Bradie and Leung 2017). This involved evaluating pairwise correlations among all potential predictor variables. Following the methodology of Sillero et al. (2021), when a correlation coefficient (*r*) exceeded 0.8, the variable with the highest Variance Inflation Factor (VIF) was systematically excluded. These calculations were performed using the usdm R package (Naimi et al. 2013). Based on this procedure and preliminary ensemble modeling assessments, the final set of variables retained for subsequent analyses comprised: Mean monthly PET of the Driest Quarter (PET of the Driest Quarter), Precipitation of Wettest Month (Bio13), Climatic Moisture Index, Mean Temperature of the Driest Quarter (Bio9), monthly variability in potential evapotranspiration (PET seasonality), and Mean Temperature of the Coldest Month.

Having confirmed that our occurrence record dataset was sufficient for developing reliable models with high predictive power and for identifying the key environmental factors shaping the species' distribution (Van Proosdij et al. 2016; Guisan et al. 2017), we further utilized the rmaxent package. This enabled us to pinpoint the environmental variables that exert the most substantial limiting effect on the species' distribution throughout Africa. The rmaxent package incorporates a limiting function (Baumgartner and Wilson 2025), which identifies the variable most responsible for reducing habitat suitability at a specific location. This function operates by calculating the decline in suitability for each predictor, relative to the suitability achieved when that predictor's value is set to the mean (or median for categorical variables) observed at occurrence sites. The predictor causing the largest drop in suitability is then identified as the most limiting factor for that environment.

### 2.5 Current Species Distribution and Climate Change Modeling Procedures

To model the species' distribution, we employed an ensemble framework, integrating both straightforward and sophisticated modeling algorithms through the biomod2 R package (Thuiller et al. 2012). Our selection of simple algorithms included Generalized Linear Models (GLM) and Flexible Discriminant Analysis (FDA), while the more complex machine learning methods comprised Generalized Boosting Models (GBM), Random Forest (RF), and Maximum Entropy. This approach was intentionally chosen to leverage the strengths of both extrapolative (simple) and interpolative (complex) models in our SDM analysis, and we utilized this ensemble modeling strategy within the *biomod2* R environment to predict areas with climatically suitable conditions for the species (Thuiller et al. 2012; Ahmadi et al. 2024). For each modeling iteration, 80% of the species occurrence records were randomly partitioned for training the models, with the remaining 20% reserved for testing their performance. This partitioning process was repeated five times, utilizing a random cross‐validation strategy. Furthermore, 5000 pseudo‐absence points were generated using the random method (Wisz and Guisan 2009), which randomly samples locations within the study area. This resulted in a total of 25 individual models for the species (5 algorithms × 5 repetitions). The parameter settings for the biomod2 package were configured using the bigboss option, which employs algorithm parameters predefined by the biomod2 development team. For the creation of ensemble models, the EMca technique was utilized. This method involves converting probability outputs from the individual models into binary (presence/absence) predictions based on established thresholds (TSS > 0.7 for this study). The final ensemble prediction was derived by averaging these binary predictions, effectively functioning as a simple voting system (Thuiller et al. 2012; Guéguen et al. 2025; See Supplemental Methods in Appendix S2).

For future projections of the species' distribution, we used Shared Socioeconomic Pathways (SSPs) SSP1‐2.6 and SSP2‐4.5 from the Coupled Model Intercomparison Project Phase 6 (CMIP6) as the core inputs for our contemporary climate models. These pathways were chosen specifically to perform an assessment of the species' resilience under more moderate climate change trajectories. SSP1‐2.6 represents a low‐emission pathway where global society shifts towards sustainable development, offering the best‐case scenario for conservation planning. SSP2‐4.5 represents a moderate‐emission pathway where socioeconomic trends follow historical patterns, serving as a critical intermediate benchmark. By focusing on these two less‐severe pathways (SSP1‐2.6 and SSP2‐4.5), the study aims to elucidate the minimum level of negative impact the species is likely to face, providing a foundation for developing resilient, forward‐looking conservation strategies (IPCC 2021). To cover the periods from 2041 to 2060 and 2080 to 2100, five distinct Global Circulation Models (GCMs) were selected for each future scenario based on their availability and desired resolution in the WorldClim database (Fick and Hijmans 2017): ACCESS‐CM2 (Dix et al. 2019), EC‐Earth3‐Veg (EC‐Earth Consortium 2020), IPSL‐CM6A‐LR (Boucher et al. 2019), MIROC6 (Shiogama et al. 2019), and MRI‐ESM2‐0 (Yukimoto et al. 2019). Integrating diverse future climate scenarios with multiple GCMs allowed us to comprehensively examine a range of potential climate trajectories and assess the inherent uncertainties of the GCMs themselves. Since reliance on a single GCM is a major factor that increases uncertainties in future projection maps (Porfirio et al. 2014), we opted to utilize an ensemble of five selected GCMs. Subsequently, the prediction outputs from the five GCMs were averaged and classified into three suitability categories using the Natural Breaks (Jenks) optimization in ArcMap v10.8 (ESRI 2025).

Model performance was rigorously evaluated using the Area Under the Receiver Operating Characteristic (ROC) curve (AUC) and the True Skill Statistic (TSS). An AUC value above 0.9 indicated excellent model performance. The TSS, which quantifies the net prediction success rate by considering both presence and pseudo‐absence data, was interpreted as follows: values between 0.40 and 0.75 indicated good performance, while values > 0.75 denoted excellent performance (Allouche et al. 2006; Araujo and New 2007; Leroy et al. 2018; Fitzpatrick et al. 2021). In developing the ensemble models, we retained only the individual models that showed a TSS value above 0.7. Ultimately, these refined ensemble models were used to project the species' potential future geographical distributions.

## 3 Results

### 3.1 Evaluation of Models' Performance

Considering the model evaluation metrics, all algorithms demonstrate excellent performance in predicting the distribution of *P. erinaceus* . The Random Forest model exhibits particularly outstanding results with an AUC of 1 and a TSS of 0.989, indicating near‐perfect discrimination and high accuracy in predicting both presence and pseudo‐absence. Gradient Boosting Machine also shows exceptional predictive power with an AUC of 0.985 and a TSS of 0.89. Furthermore, Maximum Entropy (AUC: 0.976, TSS: 0.857), Generalized Linear Model (AUC: 0.973, TSS: 0.859), and Flexible Discriminant Analysis (AUC: 0.963, TSS: 0.838) all demonstrate strong and reliable performance, further solidifying the robustness and accuracy of the species distribution models for *P. erinaceus* .

### 3.2 Current Climatic Niche and the Role of Key Factors (Temperature, Precipitation, and Evapotranspiration) in Limiting *P. erinaceus*'s Distribution in Africa

#### 3.2.1 Delineating the Current Climatic Niche and Range Suitability

**FIGURE 2:**
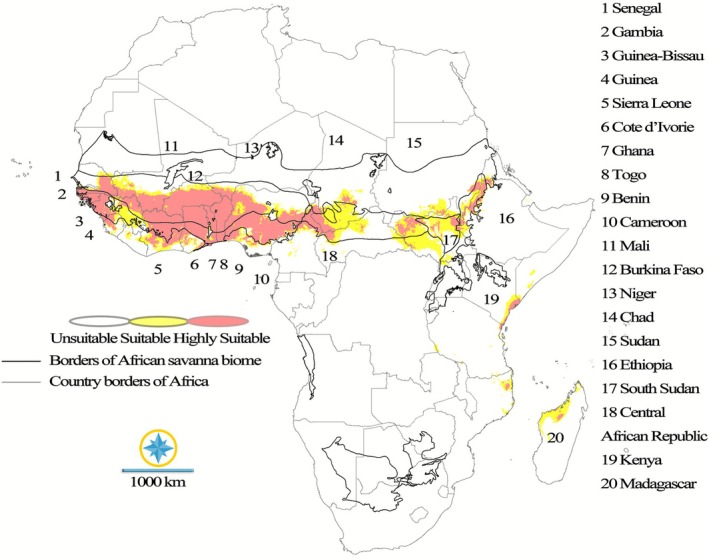
Current climatic suitability of the species in Africa, ranging from unsuitable to highly suitable areas.

The species distribution model for *P. erinaceus* revealed distinct patterns of habitat suitability across Africa. Notably, West Africa emerged as a region with significant areas of high suitability. Countries such as Senegal, Gambia, Guinea‐Bissau, Guinea, Sierra Leone, Liberia, Ivory Coast, Burkina Faso, Ghana, Togo, Benin, and the southern parts of Nigeria exhibited prominent red zones, indicating highly favorable climatic conditions conducive to dense populations and optimal growth of *P. erinaceus* . In contrast, the southern regions of Mali and Niger displayed both red and yellow zones, suggesting suitable habitats where the species can grow, albeit with potentially less vigor. Central Africa also presented a mix of habitat suitability, with parts of Cameroon, the Central African Republic, and southern Chad showing both yellow and red zones. This indicated a range of suitable to highly suitable areas within this region. In East Africa, suitable climatic conditions, represented by yellow zones, were observed in the southern regions of Sudan and South Sudan, as well as southwestern Ethiopia. Lastly, Southern Africa, specifically parts of Mozambique and Madagascar, exhibited both yellow and red zones, suggesting a gradient of suitable to highly suitable habitats. These findings highlight the variable climatic suitability for *P. erinaceus* across the African continent, emphasizing the importance of considering regional differences in conservation and management strategies (Figure 2).

The species distribution model for *P. erinaceus* revealed distinct patterns of habitat suitability across Africa. High suitability (red zones), indicative of highly favorable climatic conditions, was prominently mapped across West African countries such as Senegal, Gambia, Guinea‐Bissau, Guinea, Sierra Leone, Liberia, Ivory Coast, Burkina Faso, Ghana, Togo, Benin, and the southern parts of Nigeria. Suitable areas (yellow zones), where growth is possible but potentially less vigorous, were observed mixed with red zones in the southern regions of Mali and Niger. Central Africa (parts of Cameroon, the Central African Republic, and southern Chad) and Southern Africa (parts of Mozambique and Madagascar) also exhibited a mix of yellow and red zones, suggesting a gradient of suitable to highly suitable habitats. Finally, suitable climatic conditions (yellow zones) were found in East Africa, including the southern regions of Sudan and South Sudan, as well as southwestern Ethiopia. These findings highlight the variable climatic suitability for *P. erinaceus* across the African continent, emphasizing the importance of considering regional differences in conservation and management strategies (Figure 2).

#### 3.2.2 The Dominant Role of Temperature and Precipitation Seasonality

The analysis also revealed that the distribution of the species across Africa is predominantly constrained by specific climatic variables, with the Mean Temperature of the Driest Quarter (pink) and the Climatic Moisture Index (light green) emerging as the most influential. The Mean Temperature of the Driest Quarter exerts a substantial limiting effect across a wide latitudinal range, notably in the northern Sahara and Sahel regions, as well as parts of Southern Africa, where extreme temperatures during the driest period likely impede species establishment and survival. Similarly, the Climatic Moisture Index highlights moisture availability as a critical limiting factor, particularly in transitional arid and semi‐arid zones, including the Sahel, parts of East Africa, and the periphery of the Kalahari Desert.

**FIGURE 3:**
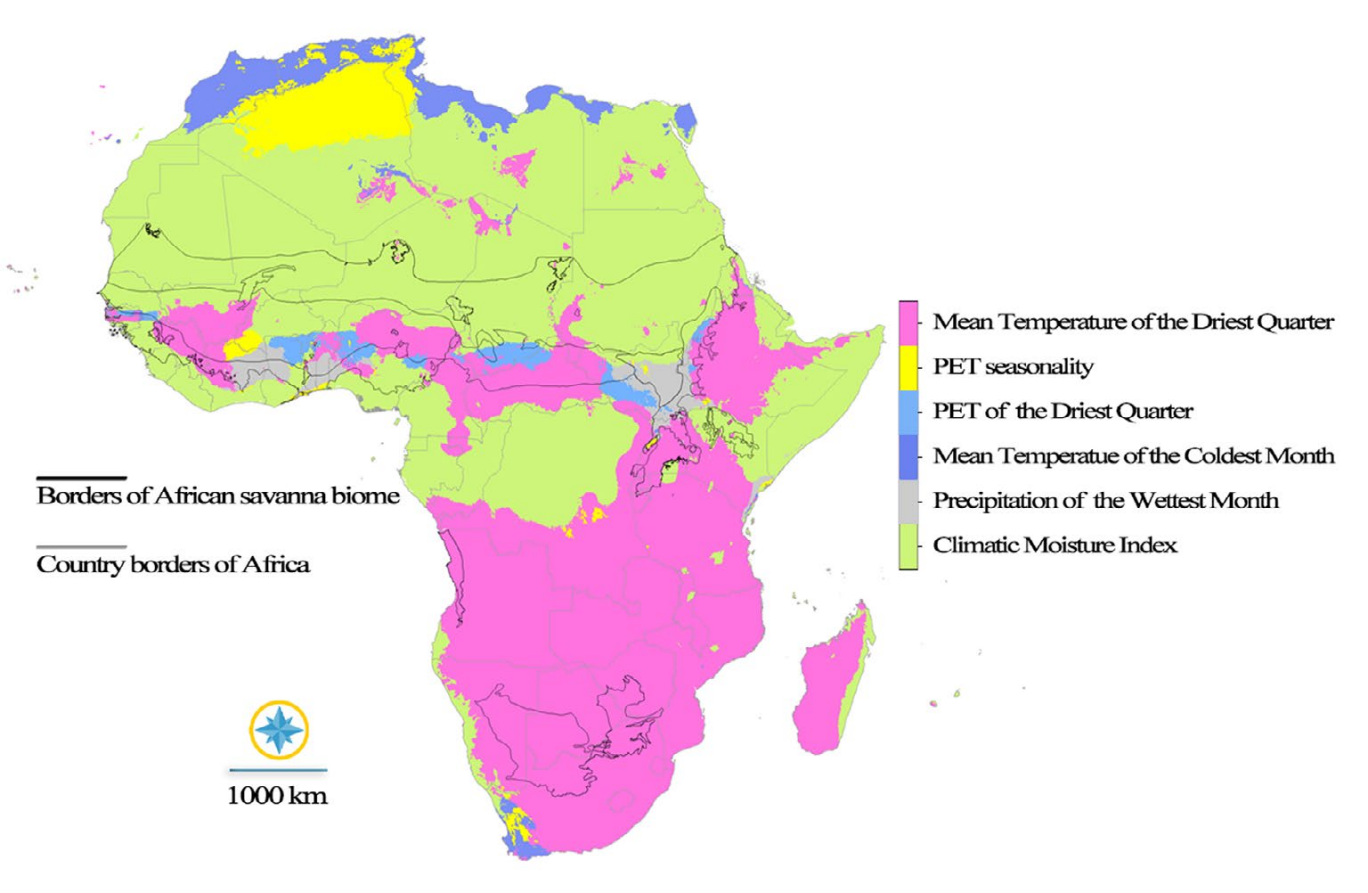
The variables that determine the current distribution suitability of the species in Africa.

While these two climate variables stand out, other climatic factors also contribute to shaping the species' distribution. The Precipitation of the Wettest Month (gray) influences species presence in regions with pronounced wet and dry seasons, such as West Africa and equatorial areas. PET seasonality (yellow) and PET of the Driest Quarter (light blue), indicators of potential evapotranspiration, are likely significant in areas experiencing high water stress, particularly in arid and semi‐arid regions. Lastly, the Mean Temperature of the Coldest Month (darker blue) appears to limit distribution in higher altitude and cooler winter regions, such as parts of Southern Africa and mountainous terrains. However, the relative importance of each variable varies regionally, reflecting the diverse climatic gradients across the continent (Figure 3).

#### 3.2.3 Water Availability and Evapotranspiration as Limiting Bioclimatic Drivers

**FIGURE 4:**
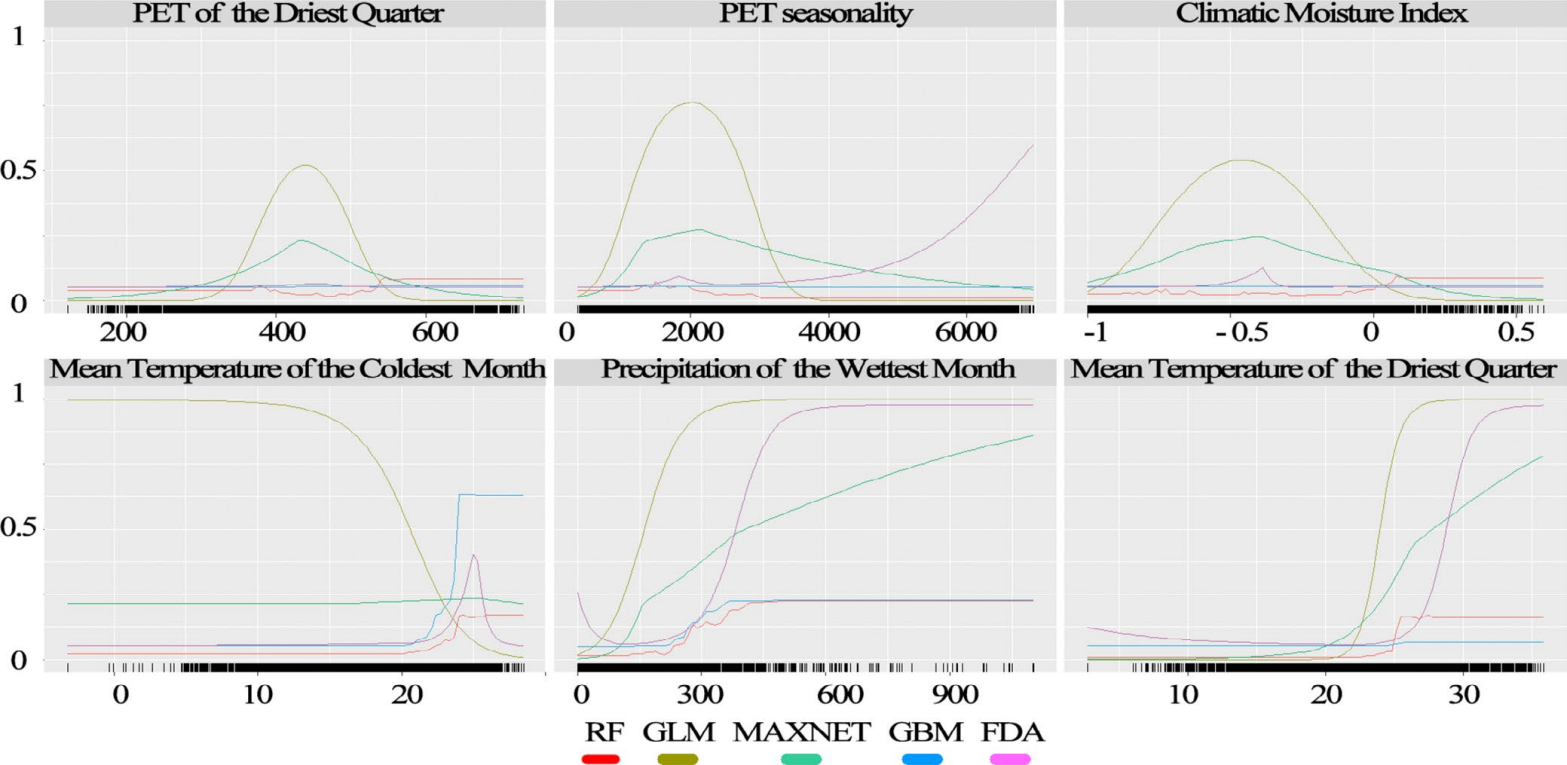
The variable responses of the species to six climatic variables in Africa.

Based on our ensemble projection, the response curves of the species to different climatic variables differ slightly (Figure 4).

PET of the Driest Quarter: Most models indicate an optimal PET value around 400, with RF showing a secondary lower suitability between 200 and 300. GLM suggests low suitability across the range, while FDA indicates a bimodal response with peaks at 300 and 500.

PET Seasonality: Several models show high suitability between 1000 and 3500, with peaks varying slightly within this range. FDA presents a more complex response with peaks around 1000, 2500, and 4500.

Climatic Moisture Index: A consistent peak in suitability around −0.2 is observed across RF, GLM, MAXENT, and GBM. FDA shows a bimodal response with peaks at −0.4 and 0.

Mean Temperature of the Coldest Month: Most models show a sharp increase in suitability around 20°C. FDA indicates a more complex response with a peak at 15°C and another increase above 20°C.

Precipitation of the Wettest Month: Generally, suitability increases with precipitation. GLM and MAXENT show increases around 300 mm, while RF and GBM show more gradual increases. FDA indicates increases around 300 and 700 mm.

Mean Temperature of the Driest Quarter: A consistent threshold effect around 25°C is observed across most models, with low suitability below and a sharp increase above this temperature.

The species exhibits varying responses to different climatic variables, as predicted by different ecological niche modeling algorithms. For the PET of the Driest Quarter, most models suggest an optimal range around 400. For PET Seasonality, the species generally prefers values between 1000 and 3000. The Climatic Moisture Index shows a peak suitability around −0.2. The Mean Temperature of the Coldest Month appears to be a threshold variable, with a sharp increase in suitability above approximately 20°C. Similarly, the Mean Temperature of the Driest Quarter also shows a sharp increase in suitability above roughly 25°C. The response to Precipitation of the Wettest Month is generally positive, with increasing suitability as precipitation increases, although the shape of this response varies across the model algorithms.

### 3.3 Future Suitable Climate Areas for the Species in Africa

#### 3.3.1 Mid‐21st Century Projections (2040–2060)

The projections for the 2040–2060 period indicate notable shifts in the climatically suitable habitat for *P. erinaceus* across Africa, with differences emerging between the low‐ and moderate‐emission pathways.

Under the SSP1‐2.6 emission scenario, the species' core habitat is largely maintained, suggesting continued favorable conditions in West Africa. Countries such as Senegal, Gambia, Guinea‐Bissau, Guinea, Sierra Leone, Liberia, Ivory Coast, Burkina Faso, Ghana, Togo, Benin, and southern Nigeria are likely to retain significant areas of high suitability (red zones). However, the projection also suggests a potential contraction of these highly suitable areas in the southern regions of Mali and Niger, where the appearance of yellow zones indicates a shift towards less optimal, though still suitable habitats. In Central Africa, areas within Cameroon, the Central African Republic, and southern Chad may experience a redistribution of suitable and highly suitable zones. East Africa (southern Sudan and South Sudan, and southwestern Ethiopia) and Southern Africa (parts of Mozambique and Madagascar) might similarly witness a redistribution in the extent of yellow and red zones, reflecting subtle shifts in climatic conditions.

**FIGURE 5:**
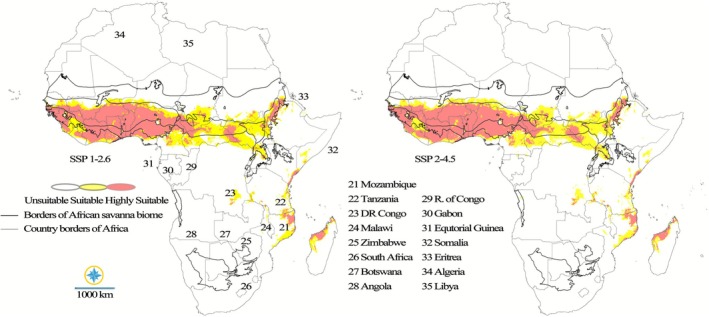
Future climatic suitability of *P. erinaceus* in Africa under the SSP1‐2.6 and SSP2‐4.5 scenarios for the 2040–2060 period.

Under the SSP2‐4.5 emissions scenario, the projected distribution shows a slightly more pronounced shift and greater uncertainty compared to the SSP1‐2.6 pathway. Countries in West Africa projected to still retain suitable and highly suitable areas will continue to do so, though the quality might decline locally. Central Africa, including parts of Cameroon, the Central African Republic, and southern Chad, may witness a substantial alteration in the balance between yellow and red zones, indicating a shift towards less favorable conditions. Notably, East Africa, encompassing southern Sudan and South Sudan, as well as southwestern Ethiopia, is expected to observe a contraction of yellow zones, suggesting reduced overall suitability. Conversely, Southern Africa, specifically areas within Mozambique and Madagascar, could see a significant change in the distribution of yellow and red zones, potentially leading to a slight increase in overall habitat suitability in these isolated patches (Figure 5).

#### 3.3.2 End‐of‐Century Projections (2080–2100)

The climatic suitability projections for *P. erinaceus* in the 2080–2100 period reveal a complex relationship between emission intensity and habitat quality and extent.

The low‐emission scenario suggests a complex reorganization of the species' suitable niche compared to its current distribution. The highly suitable core habitat, which currently forms a continuous band across the Sahel‐Savanna zones of West and Central Africa (including Senegal, Mali, Burkina Faso, and Sudan), is projected to maintain its central integrity. While this core remains the primary area for species persistence, there is evidence of slight fragmentation or contraction along its northern margins. A notable shift eastward suggests that countries like Ethiopia and parts of Sudan will retain or slightly increase their highest suitability levels. Crucially, the suitable climatic niche is predicted to undergo a substantial continental expansion. The suitable area is projected to expand dramatically southward, covering vast new territories in Southern Africa, including parts of Angola, Zambia, and Mozambique, and significantly increasing the size and connectivity of currently isolated patches in East Africa, particularly in Kenya and Madagascar. This expansion suggests that while the species' most favorable environmental conditions remain largely within its current historical range, the overall area where *P. erinaceus* could potentially establish viable populations will increase significantly, offering new possibilities for conservation and assisted migration into previously unsuitable regions under this minimum damage scenario.

**FIGURE 6:**
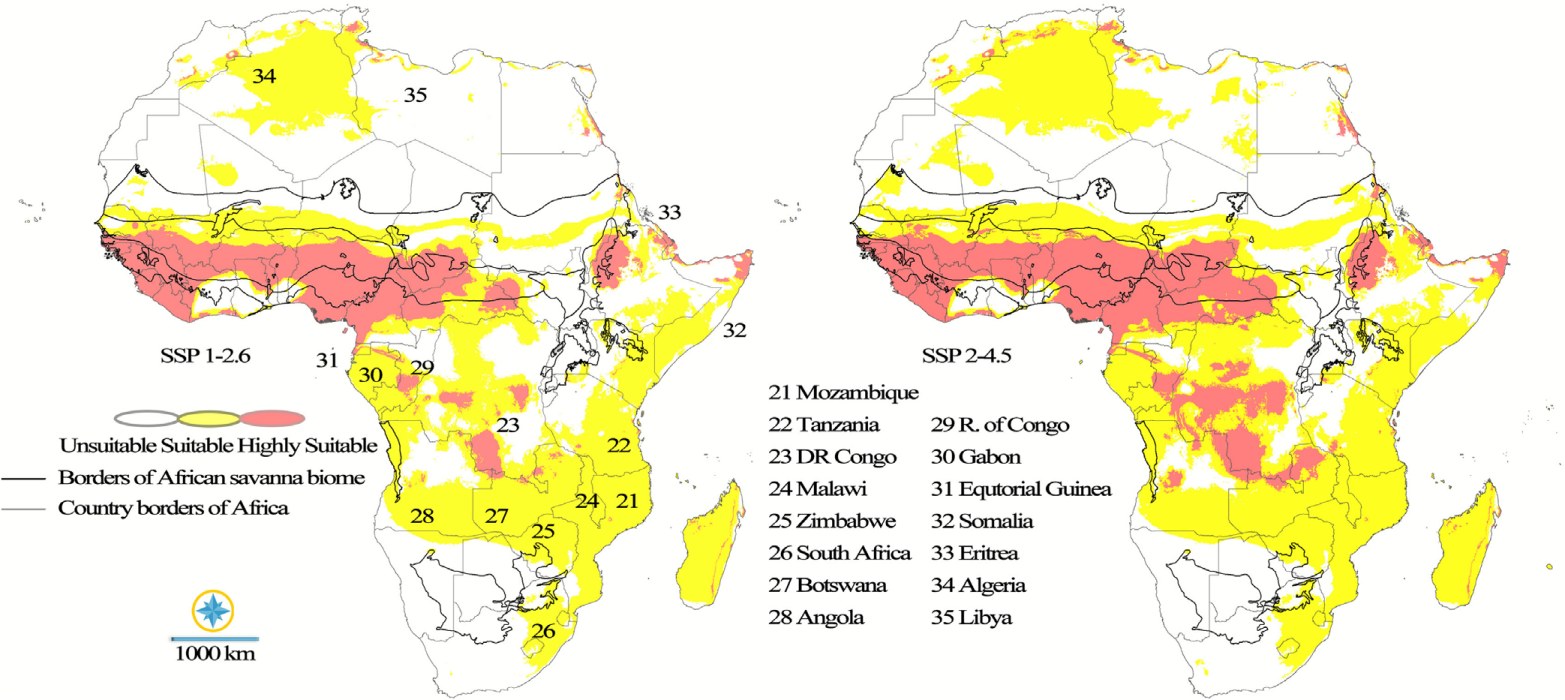
Future climatic suitability of *P. erinaceus* in Africa under the SSP1‐2.6 and SSP2‐4.5 scenarios for the 2080–2100 period.

The medium‐emission scenario suggests that a higher level of environmental stress will lead to a more pronounced contraction and fragmentation of the species' high‐quality habitat compared to the low emission scenario. Under this pathway, the highly suitable core habitat, while persisting in the main Sahel‐Savanna belt of West Africa, is projected to experience a noticeable reduction and fragmentation, particularly in the western parts (such as Senegal, Guinea‐Bissau, and Nigeria) and central areas (along the Chad/Sudan border). This indicates that even the species' most stable populations will face significant stress under increased emission levels. Furthermore, the suitable climatic areas exhibit a more limited and scattered expansion compared to the low emission scenario. Although new isolated patches of suitable habitat emerge in Southern Africa (Angola, Zambia, Mozambique) and parts of East Africa (Madagascar and certain regions of Ethiopia), these areas remain more disconnected from the main distribution belt. Overall, the mid emission scenario offers a less robust area for adaptation; the contraction of highly suitable zones, coupled with the fragmented nature of the compensatory suitable areas, underscores how increasing emissions pose a significant challenge to *P. erinaceus's* ability to maintain its existing ecological niche (Figure 6).

#### 3.3.3 Quantitative Summary of Range Dynamics

The comparison of projected climatically suitable habitat areas for *P. erinaceus* across the current time, the near‐future, and the long‐term reveals dramatic and scenario‐dependent shifts, particularly concerning the species' potential for range expansion.

Currently, the total suitable habitat (including both suitable and highly suitable areas) measures 3,191,006 km^2^ (1,237,555 km^2^ suitable and 1,953,451 km^2^ highly suitable areas). The 2050 projections show a modest expansion under both scenarios: the SSP1‐2.6 scenario sees the total suitable area increase to 4,912,175 km^2^, while the SSP2‐4.5 scenario projects a slightly greater increase to 5,244,863 km^2^.

However, the long‐term outlook for 2100 indicates a massive and unprecedented expansion of the species' potential climatic niche under both pathways. Under the SSP1‐2.6 low‐emission scenario, the total suitable area is projected to soar to 14,051,775 km^2^, representing a nearly four‐fold increase compared to the current distribution. This expansion is predominantly driven by a significant increase in the suitable category (10,455,049 km^2^) and a robust highly suitable area (3,596,726 km^2^).

The expansion is even more pronounced under the higher‐stress SSP2‐4.5 scenario, where the total suitable habitat reaches 16,645,286 km^2^—a change primarily fueled by the largest projected suitable area (11,744,029 km^2^) and the maximum projected highly suitable area (4,901,257 km^2^). This trend suggests that while the initial (2050) impacts are modest, long‐term climate change may open up vast new territories across Africa for *P. erinaceus* . The differential between the SSP scenarios highlights that the most severe emission pathway (SSP2‐4.5) paradoxically projects the largest overall climatically suitable area by 2100, underscoring the importance of considering these divergent impacts when formulating long‐term conservation and afforestation strategies.

## 4 Discussion

### 4.1 Climatic Variables and *P. erinaceus*'s Responses

Based on our research findings, the observation that most model algorithms indicate an optimal Potential Evapotranspiration of the Driest Quarter around 400 mm suggests that *P. erinaceus* favors conditions with moderate potential water loss during the driest period. The secondary low suitability within the 200–300 mm range implies a potential tolerance for drier, yet relatively humid, periods. This aligns with the species' occurrence in regions with 600 to 1500 mm of annual rainfall (Tosso 2013; Oyelowo et al. 2023) and adaptation to a moderately long dry season of 8 to 9 months (Betti 2020). Very high PET values (> 600 mm in our study) during the driest quarter may lead to prolonged excessive water stress, even considering the species' drought resistance (Asigbaase et al. 2024). Conversely, very low PET values (< 300 mm in our study) might indicate an insufficient dry period or overly humid conditions, potentially unfavorable for the species' physiology or seed dormancy breaking (Mewded et al. 2019; Amponsah et al. 2022).

The high suitability shown by several algorithms between roughly 1000 and 3500 for PET seasonality suggests that *P. erinaceus* thrives in regions with a distinct transition between wet and dry seasons. This seasonality can directly influence phenological events like leaf shedding, flowering, and fruiting (Ansah et al. 2023). While wetter conditions generally enhance tree growth (Asigbaase et al. 2024), the species also exhibits adaptations to more arid conditions (Konda et al. 2025). Therefore, similar to annual rainfall (Tosso 2013), PET seasonality may be a crucial factor in understanding its distribution. High seasonality can create distinct moisture cycles, which may facilitate the expansion and contraction of the pericarp, a mechanism that could aid in breaking dormancy for *P. erinaceus* , as observed in other species' seeds of the *Pterocarpus* Jacq. genus (Mewded et al. 2019), thereby highlighting the importance of these seasonal changes for its germination.

The consistent finding across multiple algorithms of a suitability threshold around 20°C for the Driest Quarter temperature suggests a critical physiological limit for *P. erinaceus*. Below this temperature, suitability is low, while it sharply increases above it. Despite the species' reported tolerance to a broader temperature range of 15°C–32°C and up to 40°C (Tosso 2013; Asigbaase et al. 2024; Hien et al. 2025), consistently low temperatures during the driest quarter may be metabolically insufficient for survival and basic physiological functions, potentially hindering photosynthetic rate and overall growth. Conversely, higher temperatures are associated with increased photosynthesis and biomass transfer to seeds (Wu et al. 2018; Redmond et al. 2019; Ansah et al. 2023). This 20°C threshold in the Mean Temperature of the Driest Quarter likely represents the minimum thermal requirement for *P. erinaceus* seed germination readiness, consistent with research (Adji et al. 2021; Fulgence et al. 2024) showing variable germination rates and durations across bioclimates, with suboptimal germination below this threshold and enhanced germination speed and rate above it.

In summary, *P. erinaceus* demonstrates a strong adaptation to savanna ecosystems characterized by pronounced wet and dry seasons, experiencing moderate Potential Evapotranspiration during the Driest Period, and where water availability generally slightly surpasses atmospheric demand. The identified 20°C threshold in the mean temperature of the Driest Quarter constitutes a significant bioclimatic constraint for *P. erinaceus*. Conditions exceeding this temperature appear to favor the species' physiological functions, bolster its resilience to drought stress, and provide conducive conditions for seed germination. Nevertheless, the specific mechanisms underpinning this thermal limit and its variations across different ecological zones (Kossi et al. 2020; Dimobe et al. 2022) necessitate further research. Furthermore, intraspecific variation (Biaou et al. 2023) should be considered as a potentially influential factor in the species' responses to this temperature threshold.

In addition, our analysis of the average temperature of the coldest month being around 20°C suggests a certain sensitivity of the species to low temperatures, where extreme cold may limit its distribution. The sharp increase in suitability around this threshold observed in most models aligns with the species' overall temperature tolerance (15°C–40°C) (Oyelowo et al. 2023; Asigbaase et al. 2024; Hien et al. 2025). However, the outlier may reflect local adaptations of the species across its distribution range or variations in its temperature requirements. For instance, studies have indicated differentiation in *P. erinaceus* populations across different ecological regions based on environmental variables (Biaou et al. 2023; Asigbaase et al. 2024), potentially leading to diverse suitability responses to the average temperature of the coldest month.

Considering the precipitation values of the wettest month in our study, the increase in suitability at approximately 250–300 mm suggests that this precipitation level may mark the onset of favorable conditions for the species. Supporting this, research on morphological differences has shown that trees in zones with higher annual rainfall exhibit larger morphological characteristics (Konda et al. 2025), indicating the species' adaptability to varying precipitation amounts.

Overall, within the context of present‐day climatic factors, the model outputs generally align with the known eco‐physiological characteristics of *P. erinaceus* . Minor discrepancies between models may arise from the algorithms employed and the environmental diversity within the species' broad geographical distribution. The local adaptations of the species in different ecological regions and its environmental flexibility (Biaou et al. 2023; Konda et al. 2025) may explain some of the complex responses observed in the model results.

### 4.2 Geographic Responses of *P. erinaceus* to Climate Change

Our projections for climatically suitable habitat for *P. erinaceus* for the 2040–2060 period consistently show a reduction in unsuitable areas. Both scenarios anticipate a considerable decrease in climatically unfavorable regions, accompanied by an expansion of suitable and, particularly, highly suitable areas. While the extent of this expansion differs between scenarios, the overall trend indicates a potential increase in climatically appropriate environments for the species, suggesting a degree of resilience or even a potential benefit under mid‐21st century climate change. This contrasts with previous research that often predicted solely detrimental effects (Dimobe et al. 2020; Biaou et al. 2023) and highlights the importance of considering scenario‐dependent projections in future conservation strategies for *P. erinaceus* . The realization of this potential benefit, however, is heavily influenced by the interaction of climatic changes with non‐climatic factors, such as land use (Dimobe et al. 2022; Cyrille et al. 2025), diseases, and unsustainable harvesting (Obiri et al. 2022; Biaou et al. 2023; Hien et al. 2025).

Extending this analysis to the 2080–2100 period reveals a critical divergence in habitat quality despite the projected massive overall expansion of the total climatically suitable range (reaching up to 16.6 million km^2^ under SSP2‐4.5). The low‐emission SSP1‐2.6 scenario suggests that the species' highly suitable core habitat will maintain its central integrity and continuity across the Sahel‐Savanna belt, offering robust areas for in situ conservation. Conversely, the medium‐emission SSP2‐4.5 scenario projects a more pronounced fragmentation and reduction of these highly stable, highly suitable zones, particularly along the western and northern margins of the current range. This finding suggests a paradoxical trade‐off: while higher emissions may open up more climatically suitable territory across Southern and East Africa by the end of the century, this gain is accompanied by a severe threat to the quality and connectivity of the most highly suitable core habitat, which is crucial for long‐term species persistence.

A critical consideration arising from the projected shifts—particularly under the 2080–2100 scenarios—is the displacement of significant portions of suitable and highly suitable habitat outside the boundaries of the extant Savanna biome. As previously established, as a species inherently adapted to the specific climatic, edaphic, and competitive regimes of the Sudano‐Guinean Savanna belt, the future successful colonization of these newly predicted areas poses a considerable biogeographical challenge.

While climatically suitable, these projected habitats may lack the essential non‐climatic factors, such as specific soil structures (edaphic constraints) or the vital ecological process of fire regimes typical of the Savanna, required for the establishment and long‐term persistence of *P. erinaceus* . As discussed in the introduction regarding its ecology, this is particularly relevant if the future range extension is primarily situated near the ecological margins of the West African Rainforest or Sahelian/desert biomes. Therefore, conservation strategies must move beyond purely climate‐driven suitability predictions and account for the species' ecological specificity and biome fidelity. The long‐term viability of *P. erinaceus* in the future will depend not only on climatic tolerance but also on its capacity to transcend current biome boundaries and adapt to novel ecological community dynamics.

Ultimately, while our projections indicate a potential expansion of climatically favorable habitats, they also highlight the urgent need for proactive, scenario‐dependent conservation strategies. Based on the resilience and potential range increase suggested by our findings, the immediate strategic priority for *P. erinaceus* conservation should be shifted from general climate change measures to aggressively mitigating the critical non‐climatic threats documented in the literature, such as overexploitation and unsustainable harvesting (Dumenu and Bandoh 2016; Dumenu 2019; Anthonio and Antwi‐Boasiako 2023). Addressing these persistent anthropogenic pressures is paramount for securing the species' future, irrespective of the ultimate climatic outcome.

## 5 Conclusion

This comprehensive modeling study reveals that the outcomes are highly consistent with the well‐documented ecological and physiological traits of *P. erinaceus* , thereby providing a rigorous analysis of the climatic determinants shaping the species' distribution. Furthermore, it projects potential shifts in its range in response to anticipated future climate change scenarios, offering valuable insights for conservation strategies targeting this endangered species. Therefore, to ensure the species' long‐term persistence and maintain the ecological integrity of the West African Savanna Biome, conservation strategies must prioritize aggressive, in situ actions focused on sustainable management, controlled harvesting, and the protection of current and future suitable habitats, rather than focusing solely on long‐term climate change adaptation measures. While minor discrepancies exist between models, these likely stem from differences in the algorithms used and the environmental heterogeneity across the species' wide geographic range. The species' local adaptations and environmental plasticity may account for some of the complex patterns observed in the models. Until new, more reliable mechanistic applications are derived to accurately predict species' ecophysiological responses to climatic shifts, quantifying the exact response of *P. erinaceus* to climate change remains inherently uncertain.

Regarding the species' response to future climate scenarios, our projections indicate a complex range shift by 2050, followed by a critical divergence in long‐term outcomes (2080–2100). While the total climatically viable area may expand (reaching up to 16.6 million km^2^ under SSP2‐4.5), the quality and connectivity of the core habitat are highly scenario‐dependent. Specifically, the low‐emission scenario maintains the integrity and continuity of the highly suitable core habitat, while the medium‐emission pathway projects a pronounced fragmentation and reduction of these most stable zones. Therefore, although the range may increase, the immediate strategic priority for *P. erinaceus* conservation must shift from general, long‐term climate change adaptation measures to aggressively mitigating the critical non‐climatic threats documented in the literature, such as overexploitation and unsustainable harvesting. Addressing these persistent anthropogenic pressures is paramount for securing the species' future, irrespective of the ultimate climatic outcome.

## Author Contributions

**İdris Sari:** conceptualization (equal), data curation (equal), formal analysis (equal), resources (equal), software (equal), visualization (equal), writing – original draft (equal). **Bio Ismael:** conceptualization (equal), data curation (equal), writing – original draft (equal). **Gafarou Agoundé:** data curation (equal), formal analysis (equal), resources (equal), software (equal), writing – original draft (equal). **Fazal Ullah:** writing – original draft (equal). **Faruk Yildiz:** writing – original draft (equal).

## Conflicts of Interest

The authors declare no conflicts of interest.

## Supporting information


**Appendix S1:** ece372820‐sup‐0001‐AppendixS1.xlsx.

**Appendix S2:** ece372820‐sup‐0002‐AppendixS2.docx.

## Data Availability

The occurrence data used in this study were sourced from the Global Biodiversity Information Facility. A complete list of these records is provided as a supplementary Excel spreadsheet. Additionally, the R scripts and code developed for the biomod2 species distribution modeling framework, including data preparation and analysis, are available as Supporting Information to ensure the full reproducibility of our research.
