# Supplementary material for: Climatic Variables as Drivers of Pterocarpus erinaceus (Fabaceae): Distribution and the Implications of Climate Change
Source: Ecol Evol. 2026 Jan 5;16(1):e72820. doi: 10.1002/ece3.72820 (PMC12771592; doi:10.1002/ece3.72820)
Supplement: Supplementary file 2 — Appendix S2: ece372820‐sup‐0002‐AppendixS2.docx. [file ECE3-16-e72820-s002.docx]

**Occurrences cleaning and thinning**

library(occCite);

#Creating a GBIF login

GBIFLogin <- GBIFLoginManager(user = "botanical24",

email = "botanical24@gmail.com",

pwd = "il.....24")

GBIFDownloadDirectory <- ("D:/Africa/Download directory")

mySimpleOccCiteObject <- occQuery(x = "Pterocarpus erinaceus",

datasources = c("gbif", "bien"),

GBIFLogin = GBIFLogin,

GBIFDownloadDirectory =

GBIFDownloadDirectory,

checkPreviousGBIFDownload = T)

# GBIF search results

options(max.print=999999)

head(mySimpleOccCiteObject@occResults$`Pterocarpus erinaceus`$GBIF$OccurrenceTable)

head(mySimpleOccCiteObject@occResults$`Pterocarpus erinaceus`$BIEN$OccurrenceTable)

SaveasCSV <- mySimpleOccCiteObject@occResults$`Pterocarpus erinaceus`$GBIF$OccurrenceTable

write.csv(SaveasCSV, 'D:/Africa/Download directory/PeOccs.csv')

summary(mySimpleOccCiteObject)

plot(mySimpleOccCiteObject)

**Thinning**

library(raster)

Thinning <-raster('D:/Pe/VarsAll/annualPET.tif')

par(mfrow=c(1,1))

plot(Thinning)

library(enmSdmX)

sp_occ_raw <- read.csv('D:/Pe/Occs/Pe_unthinned.csv')

sp_occ <- elimCellDuplicates(x=sp_occ_raw, r=Thinning, longLat=

c('longitude', 'latitude'))

nrow(sp_occ_raw)

nrow(sp_occ)

write.csv(sp_occ, 'D:/Pe/Occs/Pe_thinned.csv')

**VIF Analysis**

rm(list = ls())

sp_occ <- read.csv('D:/Pe/Occs/Pe_thinned.csv')

sp_occ <- as.data.frame(sp_occ)

sp_occ

library(raster)

Vars <- stack(list.files(file.path("D:/Pe/VarsAll"), pattern = 'tif$',

full.names = TRUE))

Vars <- stack(Vars)

sp_df <- as.data.frame(sp_occ)

sp_ids <- extract(Vars, cbind(sp_df$longitude, sp_df$latitude))

sp_ids <- as.data.frame(sp_ids)

head(sp_ids)

sp_ids <- extract(Vars, cbind(sp_df$longitude, sp_df$latitude))

sp_ids <- as.data.frame(sp_ids)

library(ecospat)

library(usdm)

vif(sp_ids[,37:1])

vifcor(sp_ids[,37:1], th=.8)

**Current Modeling**

rm(list = ls())

library(terra)

library(biomod2)

myData <- read.csv('D:/Pe/Occs/PeBiomod2.csv')

head(myData)

myRespName <- 'Pe'

myData <- myData[which(myData[, myRespName] == 1), ]

myResp.PA <- as.numeric(myData[, myRespName])

myRespXY <- myData[, c('X_WGS84', 'Y_WGS84')]

myExpl <- terra::rast(list.files(file.path("D:/Pe/Currentc"), pattern = 'tif$',

full.names = TRUE))

plot(myExpl)

file.out <- paste0(myRespName, "/", myRespName, ".AllModels.models.out")

if (file.exists(file.out)) {

myBiomodModelOut <- get(load(file.out))

} else {

myBiomodData.d <- BIOMOD_FormatingData(resp.var = myResp.PA,

expl.var = myExpl,

resp.xy = myRespXY,

resp.name = myRespName,

PA.nb.rep = 1,

PA.nb.absences = 5000,

PA.strategy = 'random')

myBiomodModelOut <- BIOMOD_Modeling(bm.format = myBiomodData.d,

modeling.id = 'AllModels',

models = c('RF', 'GLM', 'MAXNET', 'GBM', 'FDA'),

CV.strategy = 'random',

CV.nb.rep = 5,

CV.perc = 0.8,

OPT.strategy = 'bigboss',

metric.eval = c('TSS', 'ROC'),

var.import = 1,

seed.val = 42)

}

plot(myBiomodData.d)

get_built_models(myBiomodModelOut, full.name = NULL, PA = NULL, run = NULL, algo = NULL)

file.proj <- paste0(myRespName, "/proj_Current/", myRespName, ".Current.projection.out")

if (file.exists(file.proj)) {

myBiomodProj <- get(load(file.proj))

} else {

# Project single models

myBiomodProj <- BIOMOD_Projection(bm.mod = myBiomodModelOut,

proj.name = 'Current',

new.env = myExpl,

models.chosen = 'all',

build.clamping.mask = TRUE)

}

file.EM <- paste0(myRespName, "/", myRespName, ".AllModels.ensemble.models.out")

if (file.exists(file.EM)) {

myBiomodEM <- get(load(file.EM))

} else {

# Model ensemble models

myBiomodEM <- BIOMOD_EnsembleModeling(bm.mod = myBiomodModelOut,

models.chosen = 'all',

em.by = 'all',

em.algo = c('EMca'),

metric.select = c('TSS'),

metric.select.dataset = 'validation',

metric.select.thresh = c(0.7),

metric.eval = c('TSS', 'ROC'),

var.import = 1,

seed.val = 42)

}

myBiomodEM

# Project ensemble models (from single projections)

myBiomodEMProj <- BIOMOD_EnsembleForecasting(bm.em = myBiomodEM,

bm.proj = myBiomodProj,

models.chosen = 'all',

metric.binary = 'all',

metric.filter = 'all')

myBiomodEMProj <- BIOMOD_EnsembleForecasting(bm.em = myBiomodEM,

proj.name = 'CurrentEM',

new.env = myExpl,

models.chosen = 'all',

metric.binary = 'all',

metric.filter = 'all')

myBiomodEMProj

plot(myBiomodEMProj)

show(myBiomodEMProj)

bm_PlotEvalMean(bm.out = myBiomodModelOut)

GETSS <- get_evaluations(

myBiomodModelOut,

full.name = NULL,

PA = NULL,

run = NULL,

algo = NULL,

metric.eval = 'TSS'

)

GETSS

write.csv(GETSS, 'D:/Pe/Results/GETTSSPAPER-Pe.csv')

GEROC <- get_evaluations(

myBiomodModelOut,

full.name = NULL,

PA = NULL,

run = NULL,

algo = NULL,

metric.eval = 'ROC'

)

GEROC

write.csv(GEROC, 'D:/Pe/Results/GETROCPAPER-Pe.csv')

get_formal_data(myBiomodModelOut, subinfo = NULL)

# Get evaluation scores & variables importance

EV <- get_evaluations(myBiomodModelOut)

VI <- get_variables_importance(myBiomodModelOut)

write.csv(EV, 'D:/Pe/Results/EvalCurrent-PePAPERL.csv')

write.csv(VI, 'D:/Pe/Results/VarImpCurrent-PePAPERL.csv')

A <- bm_PlotEvalMean(bm.out = myBiomodModelOut, dataset = 'calibration')

B <- bm_PlotEvalMean(bm.out = myBiomodModelOut, dataset = 'validation')

C <- bm_PlotEvalBoxplot(bm.out = myBiomodModelOut, group.by = c('algo', 'run'))

A

B

options(max.print=25000)

C

D <- bm_PlotVarImpBoxplot(bm.out = myBiomodModelOut, group.by = c('expl.var', 'algo', 'algo'))

E <- bm_PlotVarImpBoxplot(bm.out = myBiomodModelOut, group.by = c('expl.var', 'algo', 'run'))

F <- bm_PlotVarImpBoxplot(bm.out = myBiomodModelOut, group.by = c('algo', 'expl.var', 'run'))

D

E

F

# # Represent response curves

mods <- get_built_models(myBiomodModelOut, run = 'allRun')

bm_PlotResponseCurves(bm.out = myBiomodModelOut,

models.chosen = mods,

fixed.var = 'mean')

# # Represent response curves

mods <- get_built_models(myBiomodModelOut, run = 'RUN1')

bm_PlotResponseCurves(bm.out = myBiomodModelOut,

models.chosen = mods,

fixed.var = 'mean')

**Ensemble Future Modeling**

# access126

myExplFutureF1 <- terra::rast(list.files(file.path("D:/Pe/access126c"), pattern = 'tif$',

full.names = TRUE))

myBiomodEMProjFut1 <- BIOMOD_EnsembleForecasting(bm.em = myBiomodEM,

proj.name = 'access126',

new.env = myExplFutureF1,

models.chosen = 'all',

metric.binary = 'all',

metric.filter = 'all')

myBiomodEMProjFut1

plot(myBiomodEMProjFut1)

CurrentProj <- get_predictions(myBiomodEMProj,

metric.binary = "TSS",

model.as.col = TRUE)

FutureProjF1 <- get_predictions(myBiomodEMProjFut1,

metric.binary = "TSS",

model.as.col = TRUE)

myBiomodRangeSizeF1 <- BIOMOD_RangeSize(proj.current = CurrentProj, proj.future = FutureProjF1)

myBiomodRangeSizeF1$Compt.By.Models

plot(myBiomodRangeSizeF1$Diff.By.Pixel)

# Represent main results

bm_PlotRangeSize(bm.range = myBiomodRangeSizeF1)

Diff1 <- writeRaster(myBiomodRangeSizeF1$Diff.By.Pixel, overwrite=TRUE, 'D:/Pe/Results/access126.tif')

X

X

X

X

X

X

# mri245

myExplFutureF10 <- terra::rast(list.files(file.path("D:/Pe/mri245c"), pattern = 'tif$',

full.names = TRUE))

myBiomodEMProjFut10 <- BIOMOD_EnsembleForecasting(bm.em = myBiomodEM,

proj.name = 'mri245',

new.env = myExplFutureF10,

models.chosen = 'all',

metric.binary = 'all',

metric.filter = 'all')

myBiomodEMProjFut10

plot(myBiomodEMProjFut10)

CurrentProj <- get_predictions(myBiomodEMProj,

metric.binary = "TSS",

model.as.col = TRUE)

FutureProjF10 <- get_predictions(myBiomodEMProjFut10,

metric.binary = "TSS",

model.as.col = TRUE)

myBiomodRangeSizeF10 <- BIOMOD_RangeSize(proj.current = CurrentProj, proj.future = FutureProjF10)

myBiomodRangeSizeF10$Compt.By.Models

plot(myBiomodRangeSizeF10$Diff.By.Pixel)

# Represent main results

bm_PlotRangeSize(bm.range = myBiomodRangeSizeF10)

Diff10 <- writeRaster(myBiomodRangeSizeF10$Diff.By.Pixel, overwrite=TRUE, 'D:/Pe/Results/mri245.tif')

**Averaging Future Predictions**

library(raster)

SSP 126 2050

access1262050 <- raster("proj_access126_Pe_ensemble.tif")

earthveg1262050 <- raster("proj_earthveg126_Pe_ensemble.tif")

ipsl1262050 <- raster("proj_ipsl126_Pe_ensemble.tif")

miroc1262050 <- raster("proj_miroc126_Pe_ensemble.tif")

mri1262050 <- raster("proj_mri126_Pe_ensemble.tif")

models1262050 <- stack(access1262050,

earthveg1262050,

ipsl1262050,

miroc1262050,

mri1262050)

models1262050 <- brick(models1262050)

mdl2050_126_mean <- calc(models1262050, mean)

plot(mdl2050_126_mean)

mdl2050_126_mean_tif <- writeRaster(mdl2050_126_mean, overwrite=TRUE, 'D:/Pe/Results/mdl2050_126_mean.tif')
